# Effects of *Sophora flavescens* aiton and the absorbed bioactive metabolite matrine individually and in combination with 5-fluorouracil on proliferation and apoptosis of gastric cancer cells in nude mice

**DOI:** 10.3389/fphar.2022.1047507

**Published:** 2022-11-09

**Authors:** Huan-Fu Hu, Zheng Wang, Wen-Li Tang, Xue-Ming Fu, Xiang-Jun Kong, Ying-Kun Qiu, Sheng-Yan Xi

**Affiliations:** ^1^ School of Medicine, Yueyang Vocational Technical College, Yueyang, Hunan, China; ^2^ Yueyang Key Laboratory of Comprehensive Utilization of Characteristic Chinese Herbal Medicines in Dongting Lake District, Yueyang, Hunan, China; ^3^ Department of Traditional Chinese Medicine, School of Medicine, Xiamen University, Xiamen, Fujian, China; ^4^ Department of Pharmacy, Xiang’an Hospital of Xiamen University, Xiamen, Fujian, China; ^5^ School of Pharmaceutical Sciences, Xiamen University, Xiamen, Fujian, China; ^6^ Department of Traditional Chinese Medicine, Xiang’an Hospital of Xiamen University, Xiamen, Fujian, China

**Keywords:** *Sophora flavescens* aiton, matrine, apoptosis, gastric cancer, traditional Chinese medicine

## Abstract

**Background:** *Sophora flavescens* aiton (SFA) and its main bioactive metabolite matrine are widely used in traditional Chinese medicine (TCM) preparations and have achieved good curative effects for the treatment of various tumors. However, the mechanisms underlying SFA and matrine individually and in combination with chemotherapeutic drugs for treatment of gastric cancer (GC) remain unclear.

**Aim of the study:** To elucidate the mechanisms underlying the ability of SFA and matrine individually and in combination with chemotherapeutic drugs to inhibit proliferation and promote apoptosis of human GC cells.

**Materials and methods:** Forty-eight nude mice were randomly divided into six groups that were treated with normal saline (model group), 5-fluorouracil (5-FU), SFA decoction (SFAD), matrine, SFAD+5-FU, or matrine+5-FU. A subcutaneous heterotopic tumor model was established in nude mice by implantation of human GC BGC-823 cells. All mice were treated for 28 days. Bioactive metabolites in SFA were determined by HPLC-MS/MS. The tumor volume, tumor weight, and tumor inhibition rate of mice were documented. Histopathology and ultramicroscopic pathology of tumor tissues were observed. The tumor cell cycle and apoptosis *in vivo* were detected. Serum levels of PCNA, BAX, Bcl-2, Caspase-9, Caspase-3 and cleaved Caspase-3 were measured. Protein levels of MS4A10, MS4A8, MS4A7, PCNA, BAX, Bcl-2, Caspase-3, and cleaved Caspase-3 were measured in tumor tissues.

**Results:** Both SFAD and matrine inhibited the growth of transplanted GC cells, which was more effective when combined with 5-FU. The tumor inhibition rates of the 5-FU, SFAD, matrine, SFAD+5-FU, and matrine+5-FU groups were 53.85%, 33.96%, 30.44%, 59.74%, and 56.55%, respectively. The body weight of tumor-bearing nude mice was greater in the SFAD group than the normal saline and matrine groups. SFAD+5-FU and matrine+5-FU blocked BGC-823 cells in the G_0_-G_1_/S transition, promoted apoptosis, and significantly decreased the content of serum apoptosis-inhibitory proteins (PCNA and Bcl-2) as well as protein expression of MS4A8, MS4A10, Bcl-2, and PCNA in tumor tissues, while increasing serum levels of pro-apoptotic proteins (Caspase-9, Caspase-3 and cleaved-Caspase-3) and protein expression of BAX and cleaved-Caspase-3 in tumor tissues.

**Conclusion:** SFAD and matrine both individually and in combination with 5-FU ameliorated malignancy of transplanted tumors by reducing proliferation and promoting apoptosis of BGC-823 cells. These findings confirm the anti-tumor synergistic effect of TCM and chemotherapeutic drugs.

## Introduction

Gastric cancer (GC) is the second most common malignancy of the digestive tract and accounted for 45% of all cancer deaths in China in 2020 (Cao et al., 2021). Due to the increasing incidence and poor prognosis, GC has become a major health concern worldwide (Petryszyn et al., 2020). A report by the National Cancer Center noted that mortality due to GC is two-fold higher in China than the global average. Although surgery remains the most common strategy for treatment of GC, chemotherapy and radiotherapy are widely used for management of resectable malignant tumors (Rallis et al., 2021). Antineoplastic drugs, such as 5-fluorouracil (5-FU), cytarabine, and doxorubicin, are commonly used to alleviate symptoms, control tumor growth, improve quality of life, and prolong survival. However, these drugs are expensive, toxic, and can result in drug resistance, low immune function, bone marrow suppression, and digestive dysfunction (Zhang et al., 2014). A recent study confirmed that the advantages of traditional Chinese medicine (TCM) for treatment of various cancers include lower costs, limited toxicity, improved immunity, and prolonged survival (Huang et al., 2020; Zhang et al., 2021), thereby providing a preliminary basis for the application of TCM for treatment of GC.

The chemotherapeutic agent 5-FU is an analog of uracil that acts as an antimetabolic cytotoxic drug by inhibiting thymidylate synthase and is a widely used for treatment of advanced GC (Mahlberg et al., 2017). TCM preparations combined with chemotherapeutic drugs can achieve considerable curative effects against malignant tumors (Liu et al., 2020). *Sophora flavescens* aiton (SFA) is a member of the genus *Sophora* of the family Fabaceae. Radix *Sophorae flavescentis*, known as “kushen” in Chinese, is the dried root of *S*. *flavescens* used as an important medicinal herb in TCM with a bitter taste and cold in nature applied to “clear heat”, detoxify, and “dry dampness”. In addition, kushen is often used to treat various tumors and inflammation (Xi and Gong, 2017; Li and Huang, 2019; Yu et al., 2021; Sun et al., 2022; Zheng et al., 2022). Matrine (C_15_H_24_N_2_O) is an alkaloid extracted from the dried roots and fruit of SFA with antibacterial, antiviral, antitumor, anti-inflammatory, antioxidant, sedative, analgesic, immunoregulatory, cardioprotective, and other pharmacological activities (Zhang et al., 2020). Matrine has significant inhibitory effects in a variety of tumor cells and, thus, has been applied both clinically and experimentally for treatment of various cancers of the colon, ovary, cervix, breast, thyroid, lung, and stomach (Du et al., 2018; Xie et al., 2018; Fu et al., 2020; Liu et al., 2020; Ren et al., 2020; Wang et al., 2020; Chen et al., 2021; Liang and Ju, 2021). SFA is commonly prepared as a decoction for use in clinical practice. Although matrine has been widely used in the treatment of cancer, there are no experimental and clinical reports of matrine or SFA decoction combined with small dose chemotherapeutic drugs for continuous treatment of malignant tumors.

However, it remains unclear whether a decoction of SFA has the same curative effect as matrine for treatment of tumors *in vivo* and whether a combination with 5-FU synergistically improves the effect of continuous low-dose chemotherapy for solid tumors, such as GC. Therefore, in order to simulate the reality that the clinical treatment of malignant tumor is often to receive many times of chemotherapy when the tumor can be detected and is visible to the naked eye, we designed experiments to continuously treat the nude mouse model of subcutaneous transplanted gastric cancer with matrine and SFA respectively and their combination with low-dose 5-FU. The aim of this study was to explore the efficacies of matrine and SFA combined with low-dose 5-FU for continuous treatment of GC *in vivo*.

## Materials and methods

### Study approval

The study protocol was approved by the Laboratory Animal Management and Ethics Committee of Xiamen University (approval no. XMULAC 2017-0018) and conducted in accordance with the Guide for the Care and Use of Laboratory Animals.

### GC cells

Human gastric adenocarcinoma (poorly differentiated) BGC⁃823 cells (product no. CL-0033) were purchased from Xiamen Kessel Biotechnology Co., Ltd. (Xiamen, China) and cultured in Roswell Park Memorial Institute 1640 medium supplemented with 10% fetal bovine serum (FBS), 100 U/ml of penicillin, and 100 μg/ml of streptomycin in a cell incubator at 37 °C under a humidified atmosphere of 5% CO_2_/95% air. Single layers of cells adhered to the wall of the culture dish were digested with 0.25% trypsin and cultured to a sufficient number for inoculation and modeling.

### Experimental animals

Healthy specific pathogen-free BALB/c nude mice [age, 4 weeks; male:female ratio, 1:1; mean body weight (BW), 16.0–18.0 g] were purchased from Zhejiang Vital River Laboratory Animal Technology Co., Ltd. (Jiaxing, China) [license no. SCXK (Zhe)2019-0001] and housed in the Animal Center of Xiamen University [license no. SYXK (Min)2018-0009] under a constant temperature of 22°C–26°C and relative humidity of 40%–60% with *ad libitum* access to food and water.

### Experimental drugs

SFA (product no. 190503) was purchased from Xiamen Bencao Zhenyuan Pharmaceutical Co., Ltd. (Xiamen, China) and a voucher specimen was deposited to the collection of the Xiamen Botanical Garden (Xiamen, China; http://sweetgum.nybg.org/science/ih/herbarium-details/?irn=249232; herbarium code: XMBG) for future reference. A matrine standard (20 mg/ampoule bottle, product no. SM8130) was purchased from Beijing Solarbio Technology Co., Ltd. (Beijing, China). Injectable 5-FU (250 mg/ampoule bottle, product no. 1707091) was produced by Tianjin Jin Yao Pharmaceutical Co., Ltd. (Tianjin, China). Injectable 0.9% sodium chloride (100 ml/bottle, product no. 1803152) was produced by Fujian Tianquan Pharmaceutical Co., Ltd. (Longyan, China).

### Main reagents

The following reagents were purchased in this study: RPMI-1640 medium (product no. PM150110A), FBS (product no. BS1612-105) and Annexin V-FITC/PI Apoptosis Detection Kit (specification: 50 tests, product no. A4025) were purchased from Xiamen Puning Biotechnology Co., Ltd. (Xiamen, China). Cell Cycle Detection Kit (specification: 50 tests, product no. czy2192264) was purchased from Nanjing Bencaoyuan Biotechnology Co., Ltd. (Nanjing, China). Detection kits for Enzyme-linked immunosorbent assay (ELISA): Mouse proliferating cell nuclear antigen (PCNA) ELISA kit (product no. EY-00M106501), Mouse B-cell leukemia/lymphoma 2 (Bcl-2) ELISA kit (product no. EY-00M105777), Mouse Bcl-2 associated X protein (BAX) ELISA kit (product no. EY-00M105781) and Mouse Caspase-3 ELISA kit (product no. EY-00M105566) were purchased from the Shanghai Yansheng Industrial Co., Ltd. (Shanghai, China). Mouse cleaved-Caspase-3 (D175) ELISA kit (product no. PTE-CASP3-D175-1) was purchased from the Xiamen Jiyuan Biotechnology Co., Ltd. (Xiamen, China). Mouse Caspase-9 ELISA kit (product no. GV-E12428) were purchased from the Shanghai Jiwei Biotechnology Co., Ltd. (Shanghai, China). Antibodies for Western blot analysis: Monoclonal antibodies against PCNA (product no. AC63021) and BAX (product no. AC58003) were purchased from Shanghai Acmec Biochemical Co., Ltd. (Shanghai, China). Antibodies against Bcl-2 (product no. ab59348), Caspase-3 (product no. ab32351), cleaved Caspase-3 (product no. ab2302), and MS4A10 (product no. HPA014778) were purchased from Shanghai Laizee Biotech Co., Ltd. (Shanghai, China). Antibodies against MS4A8 (product no. abx311764) and MS4A7 (product no. PA5-53603) were purchased from Beijing Biolead Biology Sci and Tech Co., Ltd. (Beijing, China) and Invitrogen Corporation (Carlsbad, CA, United States), respectively.

### Main instruments

In this study, the following instruments were employed: R105050L Double condensing rotary evaporator (Jinghua Instrument Co., Ltd., Gongyi, China), ZLGJ-18 Gland multi manifold freeze dryer (Zhengzhou Huachen Instrument Co., Ltd., Zhengzhou, China), SF2000 Electronic digital caliper (Guilin Guanglu Digital Measurement and Control Co., Ltd., Guilin, China), Thermo Scientific™ Multiskan™ GO Full wavelength microplate reader and HERAcell150i CO_2_ thermostatic cell incubator (Thermo Fisher Scientific Inc., Waltham, United States); FACS CytoFLEX Flow Cytometer (Beckman Coulter Inc., Kraemer Boulevard Brea, United States); Leica ASP6025 Automatic vacuum tissue dehydrator, Leica RM2265 automatic rotary microtome (Leica GmbH., Solms, Germany), H2500R High-speed freezing centrifuge (Xiangyi Centrifuge Instrument Co., Ltd., Changsha, China), CX41-32RFL Three-eye fluorescence microscope (Olympus Corporation, Tokyo, Japan), H-800 Transmission Electron Microscope (TEM) (Hitachi Ltd., Tokyo, Japan), Sartorius BSA623S-CW high precision analytical balance electronic scale (Sartorius Stedim Biotech GmbH, Göttingen, Germany), Bio-Rad ChemiDoc XRS + Chemiluminescence imaging system, Bio-Rad Electrophoresis and transfer system (PowerPac™ Basic electrophoresis apparatus, Mini-PREOTEAN^®^ Tetra electrophoresis chamber and Trans-Blot SD Semi-Dry Transfer Cell) (Bio-Rad Laboratories Inc., Hercules, United States), TYZD-II Horizontal shaker oscillator (Jiangsu Tianling Instrument Co., Ltd., Yancheng, China), Thermo UltiMate 3000 LC system and Thermo Q-Exactive system (Thermo Fisher Scientific, Bremen, Germany) and Cosmosil MSII-C18 column (Nakalai Tesque Co., Ltd., Kyoto, Japan).

### High-performance liquid chromatography–tandem mass spectrometry

HPLC–high-resolution electrospray ionization (HR-ESI) MS was used to analyze the effective metabolites of the SFA decoction (SFAD). The decoction was separated with a UltiMate™ 3000 HPLC system equipped with a Cosmosil MSII-C18 column (250 mm × 4.6 mm i.d., 5 μm) and coupled to a Q Exactive™ Plus Hybrid Quadrupole-Orbitrap™ Mass Spectrometer. The mobile phase was composed of acetonitrile (A) and 0.1% formic acid in water (*v*/*v*) (B), and elution was conducted in accordance with the following gradient: at 0–30 min, A from 5% to 35%, B from 95% to 65%; at 30–35 min, A from 35% to 100%, B from 65% to 0%; and at 35–45 min, A and B were retained at 100% and 0%, respectively. The column temperature was maintained at 35°C, the flow rate was 1 ml/min, and the injection volume was 5 ml. The detection wavelength of the diode array detector was set at 254 nm. The mass spectra were calibrated to *m*/*z* 100–1500 using standard mixtures of caffeine, Met-Arg-Phe-Ala, and Ultramark 1621 (Thermo Fisher Scientific) in a solution of acetonitrile-methanol containing 1% acetic acid. The mass accuracy was set at ≤5 ppm in external calibration mode with an ionization voltage of 3.5 kV and capillary temperature of 300°C.

### Drug preparation

SFA (100 g) was soaked in water (1000 ml) for 30 min and then boiled for 30 min to form a decoction, which was filtered through eight layers of degreasing gauze. The filter residue was soaked in water (800 ml), then heated for 30 min and filtered through eight layers of degreasing gauze. The filtrate was concentrated to a volume of 100 ml in a rotary evaporator at 58°C and desiccated in freeze dryer to get the extract. The weight of the final freeze-dried powder of SFA (100 g) was 13.4 g. The extraction yield was 13.4%. The freeze-dried powder was sealed and stored at 4°C. When needed, the freeze-dried SFA powder was rehydrated at a concentration of 20 mg/ml (the corresponding dosage was 0.2 g/kg) [equal to 1.5 g (dried medicinal herb)/kg, about 10 times the equivalent human dosage] and stored at 4°C for future use. Matrine (molecular weight: 248.36) was dissolved in double-distilled water to a concentration of 4.03 MMol/L (2 mg/ml, equivalent to 20 mg/kg) according to the conversion formula 1 mg/ml = (1000/molecular weight) mmoL/L. Injectable 5-FU was diluted with 0.9% normal saline and the concentration was adjusted to 0.5 mg/ml (equivalent to 10 mg/kg).

### Establishment of a transplanted tumor model, animal groupings, and drug administration

BGC-823 cells in the logarithmic growth period were digested with 0.25% trypsin−0.2% ethylenediamine tetraacetic acid-D-Hanks solution, centrifuged at 1000 rpm, and reconstituted into 6 × 10^7^/ml single-cell suspensions, which were stored on ice until used. BALB/c nude mice were anesthetized with 3% isoflurane and subcutaneously inoculated with 0.2 ml of the cell suspension (about 1.2 × 10^7^ cells). Seven days after inoculation, the mouse model was assessed in accordance with the method described by Qiao et al. (2019). Forty-eight tumor-bearing nude mice were randomly assigned to one of six groups (*n* = 8/group) that were treated with normal saline, 5-FU, SFAD, matrine, SFAD+5-FU, or matrine+5-FU. All treatments were initiated on day 2. Before treatment, the BW of each mouse was recorded. The model group was administered 0.9% normal saline by gavage at 0.1 ml/10 g BW daily. Mice in the 5-FU group were intraperitoneally injected with 0.5 mg/ml (10 mg/kg) of 5-FU at 0.1 ml/10 g BW daily. The daily dosage of SFA for human adults is 1.2 g (equal to 9 g dried medicinal herb) (Xi and Gong, 2017). Therefore, considering that the BW of an adult human is 60 kg, the adult human dosage is 0.02 g/kg and the equivalent dose for mice is about 10 times greater, which is 0.2 g/kg (Blanchard and Smoliga, 2015). Therefore, the SFAD group was administered 20 mg/ml (0.2 g/kg) of SFAD by gavage at 0.1 ml/10 g BW daily. The matrine group was administered 4.03 mmoL/L (2 mg/ml, 20 mg/kg) of matrine solution by gavage at 0.1 ml/10 g BW daily. The SFAD+5-FU group was administered 20 mg/ml of SFAD by gavage plus 0.5 mg/ml of 5-FU by intraperitoneal injection daily. The matrine+5-FU group was administered 4.03 mmoL/L (2 mg/ml, 20 mg/kg) of matrine solution by gavage plus 0.5 mg/ml of 5-FU by intraperitoneal injection daily. The mice in all groups were treated for 28 days. The general condition of the mice, including BW, physical activity, mental state, reaction ability, appetite, and urine and feces production, were monitored daily.

### Observation of general condition and determination of tumor volume, tumor weight, and inhibition rate of the transplanted tumor

General conditions (i.e., physical activity, mental state, reaction ability, appetite, urination, and defecation) were observed daily. The longest diameter 1) and the shortest diameter 2) of the subcutaneous transplanted tumors in nude mice were measured with a digital caliper (Guilin Guanglu Digital Measurement and Control Co., Ltd., Guilin, China) once per week and the tumor volume (V) was calculated as V (mm^3^) = 1/2 × a (mm) × b^2^ (mm^2^). The day after drug discontinuation, retro-orbital blood samples were collected into capillary tubes and stored at −80°C until assayed. The nude mice were killed by cervical dislocation and the transplanted tumors were collected. The tissue fluid was absorbed with filter paper and weighed. Fresh tissues were either assayed immediately or stored at −80°C for future use. The tumor inhibition rate (IR) was calculated as IR (%) = (average tumor weight of the model control group—average tumor weight of the experimental group)/(average tumor weight of the model control group) ×100%.

### Observation of tumor tissue by light microscopy and transmission electron microscopy

Fresh tumor tissue was fixed in 4% paraformaldehyde phosphate buffer for 48 h, then embedded in paraffin, sectioned, and dewaxed for 5 min in xylene, followed by water-free ethanol for 5 min, 95% ethanol for 2 min, 80% ethanol for 2 min, 70% ethanol for 2 min, and distilled water for 2 min. Then, the sections were dyed with hematoxylin dye solution for 20 min, rinsed with tap water, differentiated with differentiation fluid for 30 s, soaked in warm water for 5 min at 50°C, placed in eosin dye solution for 2 min, soaked in tap water for 5 min, then rinsed twice with 95% ethanol for 1 min, twice with anhydrous ethanol for 1 min, xylene carbolic acid (3:1) for 1 min, twice with xylene for 1 min, and sealed with neutral gum. Then, the pathological conditions of sections of each group were graded under an optical microscope. Meanwhile, the tumor tissue was fixed in 2.5% glutaraldehyde and 1% osmic acid for 2 h, respectively, dehydrated with acetone, embedded in paraffin, and cut into ultrathin sections, which were stained with uranium acetate (UA) and lead citrate (LC). Finally, the ultrastructure of the tumor cells was observed with a transmission electron microscope.

### Detection of tumor cell cycle and apoptosis by flow cytometry

At the end of 28 days of treatment, tumor tissue (about the size of a match head) was cut from each sample, ground in normal saline (0.9%), and filtered through a 300-mesh sieve to remove residual tissue. Cell suspensions were centrifuged for 5 min at 1000 rpm. After the supernatant was discarded, the cell pellet was resuspended in phosphate-buffered saline (PBS) containing 0.1% FBS, centrifuged for 5 min at 1000 rpm, and washed twice. The resulting pellet was resuspended in PBS containing 0.1% FBS, fixed with 70% ice-cold ethanol, and cooled overnight at 4°C to prepare single-cell suspensions of GC tissue. The next day, the cell suspension was centrifuged for 5 min at 1000 rpm and 4°C and washed once with PBS. The cells were resuspended in 400 μL of PBS. Then, 20 μL of RNase A solution were added and the cell suspension was incubated for 30 min at 37°C, filtered through a 400-mesh sieve, gently mixed with 400 μL of propidium iodide (PI) staining solution, incubated at 4°C in the dark for 30 min, and assayed by flow cytometry within 2 h to determine the stage of the tumor cell cycle. Meanwhile, the tumor cells were resuspended in 100 µL of 1× binding buffer. Following the addition of 5 µL of Annexin V-fluorescein isothiocyanate and 5 µL of PI staining solution, the cells were gently mixed and reacted without light at room temperature for 10 min. Finally, 400 µL of 1 × binding buffer were added and the cell suspension was mixed well. Apoptosis of tumor cells was detected by flow cytometry within 1 h with the use of a Cell Cycle Detection Kit.

### Serum levels of PCNA, BAX, Bcl-2, Caspase-9, Caspase-3, and cleaved Caspase-3

Serum proteins were detected with commercial enzyme-linked immunosorbent assay (ELISA) kits. Frozen peripheral blood samples were thawed at room temperature over a period of 30 min and centrifuged for 15 min at 3000 rpm and 4°C. Afterward, the sediment was discarded and the supernatant was collected. All reagents and standards were warmed to room temperature over a period of 30 min. Standards (100 μL) and samples (100 μL) were added to the enzyme-coated wells of a microtiter plate and incubated for 2 h at 37°C. Then, biotin-labeled antibodies against PCNA, Bax, Bcl-2, Caspase-9, Caspase-3, and cleaved Caspase-3 were added to the appropriate wells and the plate was incubated for 1 h at 37°C, washed three times with washing buffer, and then dried. Afterward, horseradish peroxidase-labeled secondary antibodies were added to the wells and the plate was incubated at 37°C for 1 h. After discarding the contents of the wells, the plate was washed with washing buffer and dried. Then, substrate solution was added to each well for 15–30 min at 37°C away from light, then discarded. Finally, stop solution was added to each well to terminate the reaction. Within 5 min, the optical density at 450 nm of each well was determined with a microplate reader to determine the actual concentration of each sample. Each sample was tested in triplicate.

### Western blot analysis of MS4A10, MS4A8, MS4A7, PCNA, BAX, Bcl-2, Caspase-3, and cleaved Caspase-3 in tumor tissues

Tumor tissue samples were lysed in radio immunoprecipitation assay (RIPA) lysate buffer on ice for 1 h. Then, the lysate was transferred into a centrifuge tube and centrifuged for 10 min at 12000 rpm and 4°C. The protein content in the supernatant was quantified with the bicinchoninic acid assay, adjusted to 2 g/L with RIPA buffer, mixed with 5 × loading buffer (4:1 v/v), and denatured in boiling water for 10 min. The proteins were then transferred to the wells of a 15% polyacrylamide gel and separated by electrophoresis at 100 V for 1 h and then at 130 V until the loading dye reached the end of the gel. Afterward, the separated proteins were transferred to a polyvinylidene fluoride membrane under the conditions of 25 mmoL/L trisbase, 192 mmoL/L glycine, transfer buffer with 20% methanol, and 100 V stabilized voltage for 60 min at 4°C. Then, the membrane was blocked with 5% skimmed milk powder at room temperature for 1 h and probed with primary antibodies [diluted in 5% bovine serum albumin with Tris-buffered saline/Tween 20 (TBST)] against glyceraldehyde-3-phosphate dehydrogenase (GAPDH) (1:1000), MS4A10 (1:1000), MS4A8 (1:1000), MS4A7 (1:1000), PCNA (1:2000), Bax (1:1000), Bcl-2 (1:1000), Caspase-3 (1:800), and cleaved Caspase-3 (1:800), while shaking for 15 min at 37°C and then overnight at 4°C. The next day, the membrane was washed three times with TBST for 5 min to remove the residual primary antibodies, then probed with horseradish peroxidase-labeled secondary antibodies diluted to 1:5000 at room temperature for 2 h and finally washed three times with TBST for 3 min. The protein bands were visualized using electrochemiluminescence reagent and imaged with a gel imaging system. The relative expression level of each target protein was calculated by dividing the gray value of the sample by that of the internal reference (GAPDH) using Image Pro-Plus 6.0 software (Media Cybernetics, Inc., Rockville, MD, United States).

### Statistical analysis

Data are expressed as the mean ± standard deviation (SD). Data were analyzed using Graph Pad Prism 8.0 statistical software (GraphPad Software, Inc., La Jolla, CA, United States). Groups were compared by one-way analyses of variance (ANOVA). Pairwise comparisons were conducted with the least significant difference test. A probability (*p*) value of <0.05 was considered statistically significant.

## Results

### Analysis of SFA decoction

**FIGURE 1:**
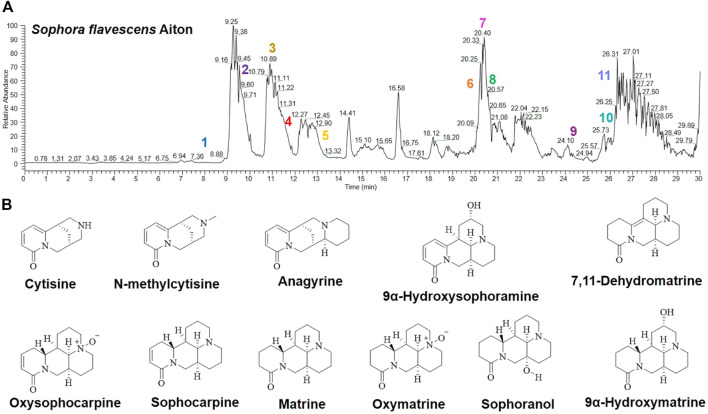
Analysis of SFAD by HPLC-MS. **(A)** HPLC-MS chemical fingerprint of SFAD. **(B)** Chemical structures of the major identified metabolites in SFAD by HPLC-MS: oxysophocarpine (1), N-methylcytisine (2), cytisine (3), sophoranol (4), oxymatrine (5), sophocarpine (6), 9α-hydroxymatrine (7), anagyrine (8), 9α-hydroxysophoramine (9), 7,11-dehydromatrine (10), and matrine (11).

As shown in Figure 1 and Table 1, the metabolites of SFA were separated by HPLC-MS and a chromatographic fingerprint was established (Figure 1). Comparisons of the retention time (RT) and the UV and MS spectra of reference samples identified the following 11 main metabolites of SFA: oxysophocarpine (peak 1, RT = 8.05 min), N-methylcytisine (peak 2, RT = 9.53 min), cytisine (peak 3, RT = 10.89 min), sophoranol (peak 4, RT = 11.47 min), oxymatrine (peak 5, RT = 13.20 min), sophocarpine (peak 6, RT = 20.17 min), 9α-hydroxymatrine (peak 7, RT = 20.40 min), anagyrine (peak 8, RT = 20.84 min), 9α-hydroxysophoramine (peak 9, RT = 24.17 min), 7,11-dehydromatrine (peak 10, RT = 26.03 min), and matrine (peak 11, RT = 26.29 min).

**TABLE 1:** ESI ion peaks elucidated from the HPLC-MS spectra of the SFA decoction.

Herb	No.	Compound	FW	MW	ESI ion peaks in herb	
					ESI^+^*	tR (min)
SFA	3	Cytisine	C_11_H_14_N_2_O	190	191.1181	10.89
	2	N-methylcytisine	C_12_H_16_N_2_O	204	205.1337	9.53
	8	Anagyrine	C_15_H_20_N_2_O	244	245.1650	20.84
	9	9α-Hydroxysophoramine	C_15_H_20_N_2_O_2_	260	261.1598	24.17
	6	Sophocarpine	C_15_H_22_N_2_O	246	247.1805	20.17
	10	7,11-dehydromatrine	C_15_H_22_N_2_O	246	247.1806	26.03
	1	Oxysophocarpine	C_15_H_22_N_2_O_2_	262	263.1756	8.05
	11	Matrine	C_15_H_24_N_2_O	248	249.1962	26.29
	5	Oxymatrine	C_15_H_24_N_2_O_2_	264	265.1914	13.20
	4	Sophoranol	C_15_H_24_N_2_O_2_	264	265.1913	11.47
	7	9α-Hydroxymatrine	C_15_H_24_N_2_O_2_	264	265.1910	20.40

*[M + H]^+^.

### Effects of SFA/matrine+5-FU on general conditions, BW change, tumor weight, and tumor volume of BGC-823-bearing nude mice

Visual observation found that mice in the normal saline (model) group exhibited good mental states, fast tumor growth, generally normal physical activity, good appetite and responsiveness, and normal urination and defecation, while those in the SFAD and matrine groups exhibited good mental states, normal physical activity and reactions, good appetite, normal urination and defecation, but slight drowsiness and slow tumor growth as compared with the model group. The tumor growth rate was relatively slower in the SFAD+5-FU and matrine+5-FU groups as compared with the model, SFAD, and matrine groups, while the mental state and activity were normal, but physical activity, reactions, and feeding were reduced, with slightly sticky stools and obvious drowsiness. Tumor growth was slowest in the 5-FU group and the mice were listless with lower body temperatures, poor physical reactions, obvious lethargy, low mobility, and poor appetite.

**FIGURE 2:**
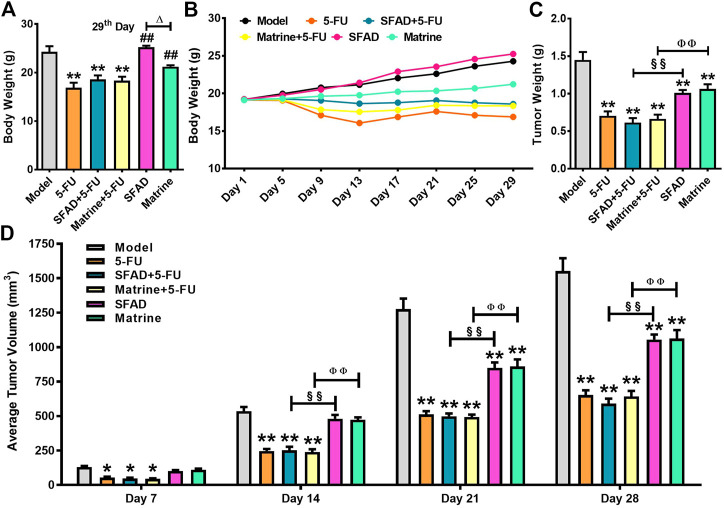
Effects of SFA/matrine+5-FU on BW, average tumor weight, and tumor volume of BGC-823-transplanted nude mice. **(A)** Average BW (g) on day 29. **(B)** Changes in BW (g) over the 29-day trial period. **(C)** Average tumor weight (g) on day 29. **(D)** Average tumor volume (mm^3^) on days 7, 14, 21, and 28. Data are presented as the mean ± SD of eight nude mice. *p < 0.05, **p < 0.01 vs*.* the model group; ^##^ *p* < 0.01 vs*.* the 5-FU group; ^△^ *p* < 0.05 for the SFAD vs. matrine groups; ^§§^ *p* < 0.01 for the SFAD+5-FU vs. SFAD groups; ^ΦΦ^ *p* < 0.01 for the matrine+5-FU vs. matrine groups (one-way ANOVA).

As compared with the model group, the BW of mice in the SFAD and matrine groups had slowly increased. Interestingly, BW was greater in the SFAD group than the matrine group (*p* < 0.05, Figure 2A), while gradually decreasing in the 5-FU, SFAD+5-FU, and matrine+5-FU groups. Notably, BW loss was lower in the SFAD+5-FU and matrine+5-FU groups than the 5-FU group (Figure 2B). Tumor volume was measured daily from 7 days post inoculation. Tumor volume had increase with time across all groups, but to different extents, with the largest increase in the model group. Comparatively, tumor volume and weight were lower in the SFAD+5-FU and matrine+5-FU groups than the SFAD and matrine groups (*p* < 0.01, Figures 2C,D). The tumor inhibition rates of the 5-FU, SFAD, matrine, SFAD+5-FU, and matrine+5-FU groups were 53.85%, 33.96%, 30.44%, 59.74%, and 56.55%, respectively.

### Effects of SFA/matrine+5-FU on tumor pathology

**FIGURE 3:**
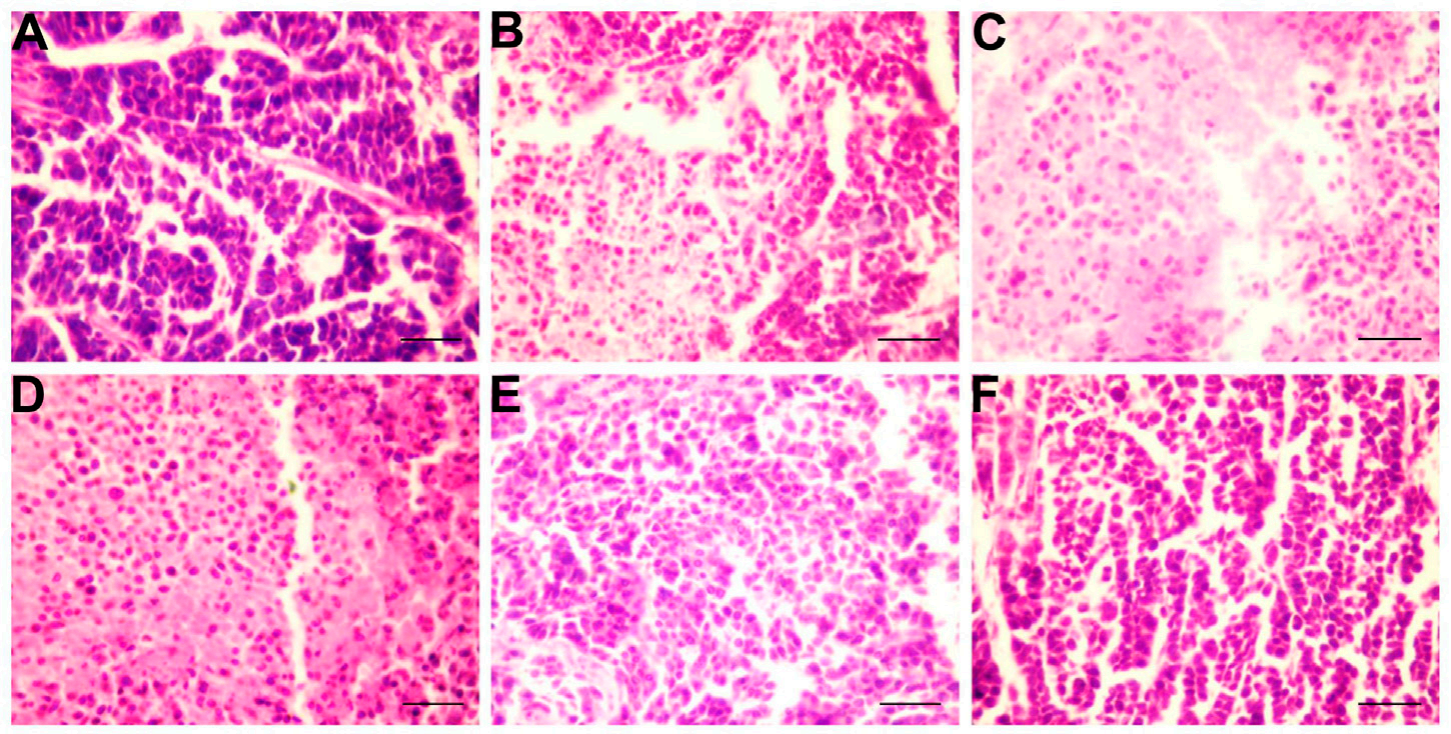
Effects of SFA/matrine+5-FU on pathological changes to tumor tissue in the BGC-823-transplanted nude mice. Sections were stained with H&E and viewed at a magnification of ×400. Scale bar = 50 μm. **(A)** Model control (negative control). **(B)** 5-FU control (0.01 g/kg) (positive control). **(C)** SFAD (0.2 g/kg)+5-FU (0.01 g/kg). **(D)** Matrine (4.03 mmoL/L)+5-FU (0.01 g/kg). **(E)** SFAD (0.2 g/kg). **(F)** Matrine (4.03 mmoL/L).

Staining with hematoxylin and eosin (H&E), as observed under a light microscope, showed that the nuclei of BGC-823 cells in the model group were large with hyperchromatism, relatively more pathological mitotic figures, large amount of nucleoplasm, and significant malignant proliferation, with large numbers of cancer cells closely arranged forming cord- or nest-like structures with vigorous growth (Figure 3A). As compared with the model group, the SFAD, 5-FU, and TCM combined chemotherapy groups showed different degrees of necrosis and apoptosis of BGC-823 cells, decreased pathological mitotic figures, significantly reduced cell density, scattered cell distribution, and increased vacuoles, especially in the combined chemotherapy groups (Figures 3B–F). Apoptosis of BGC-823 cells was most obvious in the SFAD+5-FU group, as the number of tumor cells was decreased significantly, with more dispersed distributions and fewer pathological mitotic figures (Figure 3C).

### Effects of SFA/matrine+5-FU on ultramicropathology and apoptosis of transplanted tumor cells

**FIGURE 4:**
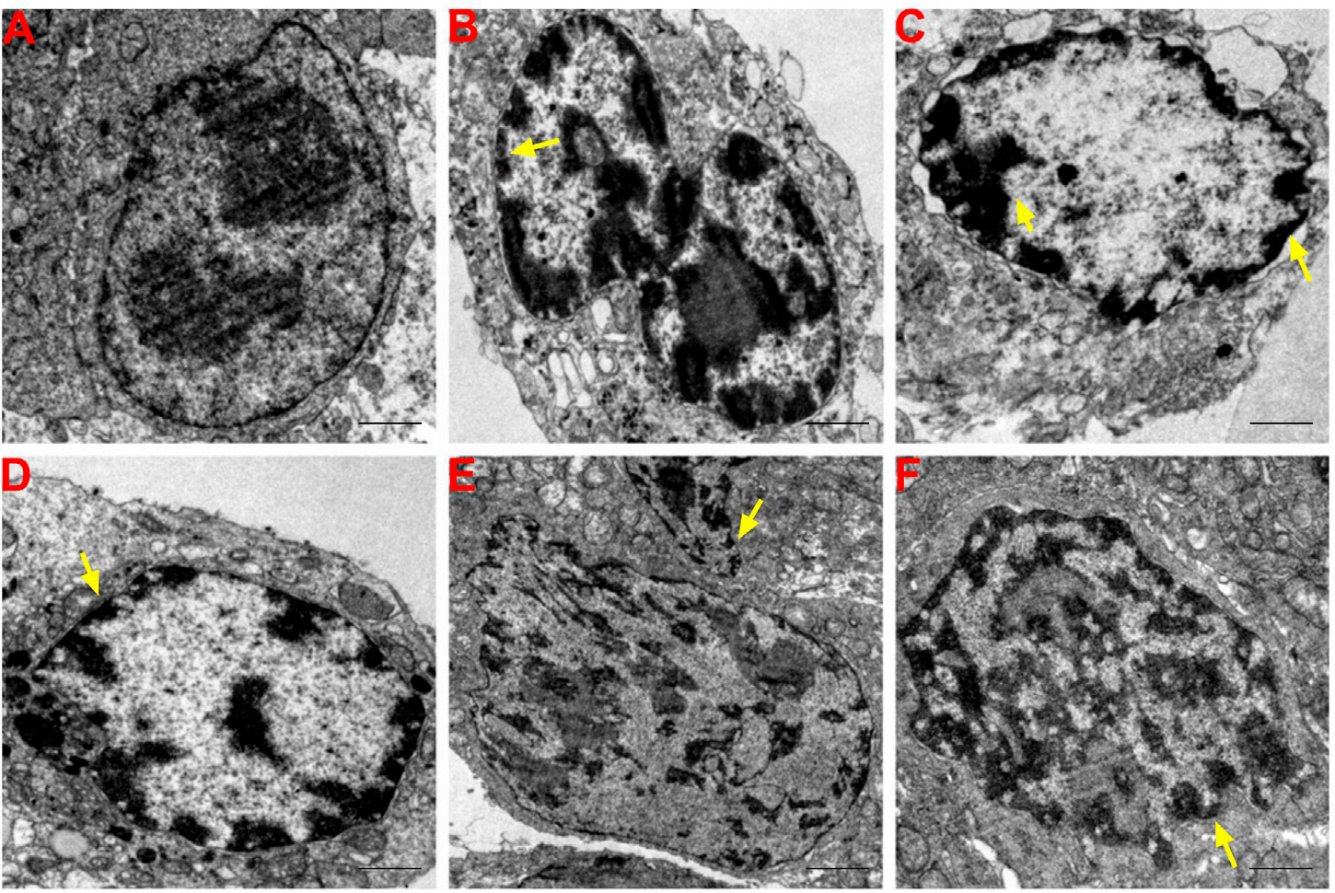
Effects of SFA/matrine+5-FU on ultramicropathological changes and apoptosis of tumors in BGC-823-transplanted nude mice. Sections were stained with UA and LC and viewed with a transmission electron microscope at a magnification of ×8000. Scale bar = 2 μm. Yellow arrows indicate nuclear pyknosis and cell membrane destruction. **(A)** Model control (negative control). **(B)** 5-FU control (0.01 g/kg) (positive control). **(C)** SFAD (0.2 g/kg)+5-FU (0.01 g/kg). **(D)** Matrine (4.03 mmoL/L)+5-FU (0.01 g/kg). **(E)** SFAD (0.2 g/kg). **(F)** Matrine (4.03 mmoL/L).

The nucleoli of BGC-823 cells were notably larger in the normal saline group and two nucleoli were observed in some tumor cells with irregular, but relatively intact, nuclear membranes. The proportion of nucleoplasm was slightly higher and chromatin in the nucleus was evenly distributed with more euchromatin and less heterochromatin distributed along the nuclear membrane (Figure 4A). Electron density of many tumor cells was relatively greater in the SFAD, matrine, 5-FU, SFAD+5-FU, and matrine+5-FU groups than the normal saline group. Chromatin in the nucleus exhibited an irregular morphology and concentrated to the edge. Many nuclear membranes were incomplete with unclear boundaries, nucleolar atrophy, chromatin shrinkage, nuclear fragmentation, and formed apoptotic bodies (Figures 4B–F).

### Effects of SFA/matrine+5-FU on the tumor cell cycle

**FIGURE 5:**
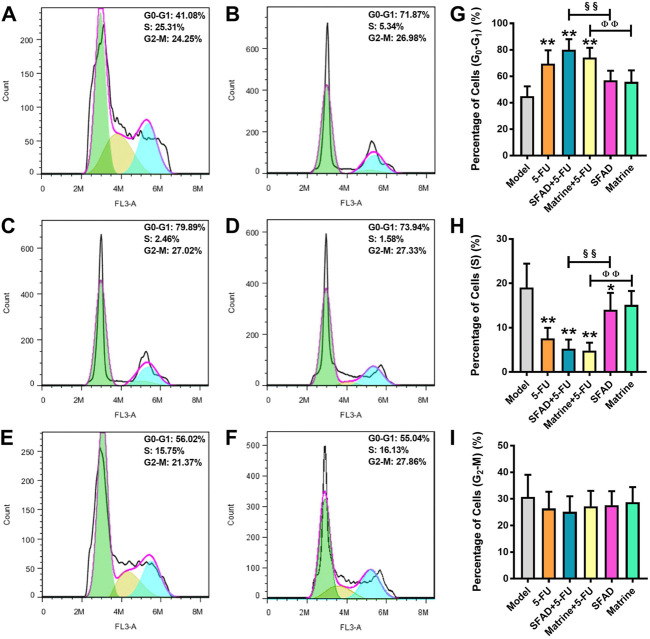
Effects of SFA/matrine+5-FU on the tumor cell cycle in BGC-823-transplanted nude mice. **(A)** Model control (negative control). **(B)** 5-FU control (0.01 g/kg) (positive control). **(C)** SFAD (0.2 g/kg)+5-FU (0.01 g/kg). **(D)** Matrine (4.03 mmoL/L)+5-FU (0.01 g/kg). **(E)** SFAD (0.2 g/kg). **(F)** Matrine (4.03 mmoL/L). **(G)** Proportion of tumor cells in the G_0_-G_1_ stage (gap phase). **(H)** Proportion of tumor cells in the S stage (synthesis phase). **(I)** Proportion of tumor cells in the G_2_-M stage (gap phase 2-mitotic phase). Data are presented as the mean ± SD of eight nude mice. *p < 0.05, **p < 0.01 vs. the model group; ^§§^ *p* < 0.01 for the SFAD+5-FU vs. SFAD groups; ^ΦΦ^ *p* < 0.01 for the matrine+5-FU vs. matrine groups (one-way ANOVA).

As shown in Figures 5A–D, single staining with PI, as detected by flow cytometry, showed that the percentage of tumor cells in the G_0_-G_1_ phase was significantly higher in the SFAD+5-FU, matrine+5-FU, and 5-FU groups than the normal saline group (*p* < 0.05, Figure 5G). Meanwhile, the percentage of tumor cells in the S phase was obviously lower in the SFAD, 5-FU, and SFAD/matrine+5-FU groups than the normal saline group (*p* < 0.05, Figures 5E,F,H). The therapeutic effects of SFAD+5-FU and matrine+5-FU were superior to those of SFAD and matrine separately (*p* < 0.01, Figures 5G,H). The number of tumor cells in the G_2_-M phase was relatively decreased in each group treated with SFAD, matrine, and 5-FU as compared with the model control group, but these differences were not significant (Figure 5I).

### Effects of SFA/matrine+5-FU on the apoptotic index of transplanted tumor cells

**FIGURE 6:**
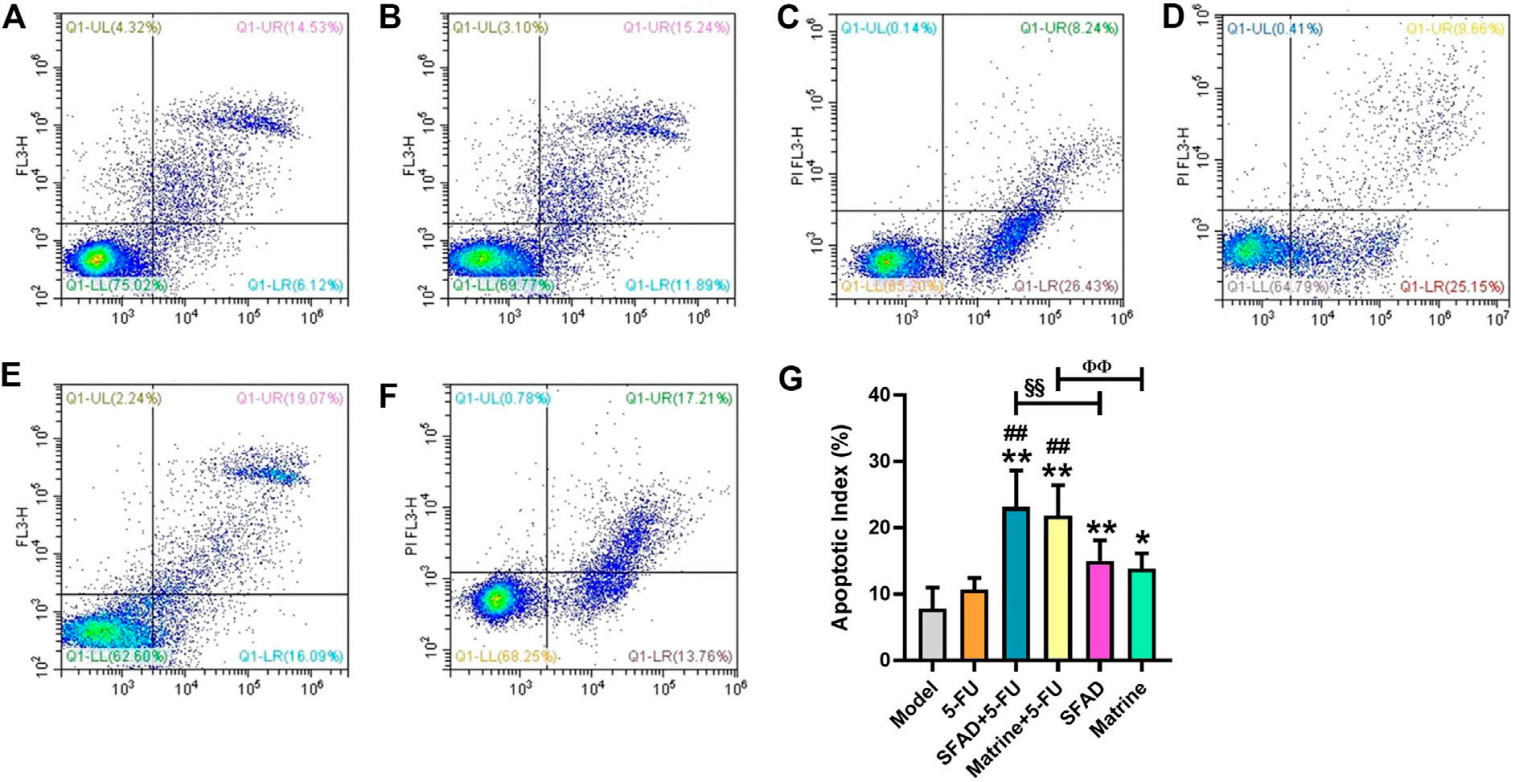
Effects of SFA/matrine+5-FU on the apoptotic index of tumor cells in BGC-823-transplanted nude mice. **(A)** Model control (negative control). **(B)** 5-FU control (0.01 g/kg) (positive control). **(C)** SFAD (0.2 g/kg)+5-FU (0.01 g/kg). **(D)** Matrine (4.03 mmoL/L)+5-FU (0.01 g/kg). **(E)** SFAD (0.2 g/kg). **(F)** Matrine (4.03 mmoL/L). **(G)** Apoptotic index of tumor cells (%). Data are presented as the mean ± SD of eight nude mice. *p < 0.05, **p < 0.01 vs. the model group; ^##^ *p* < 0.01 vs. the 5-FU group; ^§§^ *p* < 0.01 for the SFAD+5-FU vs. SFAD groups; ^ΦΦ^ *p* < 0.01 for the matrine+5-FU vs. matrine groups (one-way ANOVA).

As shown in Figure 6, the results of Annexin-V/PI staining, as detected by flow cytometry, demonstrated significantly higher apoptosis rates (apoptotic index, %) of early BGC-823 cells in the SFAD, matrine, SFAD+5-FU, and matrine+5-FU groups as compared with the normal saline, SFAD, and matrine groups (*p* < 0.05) (Figures 6A–E,G). In addition, the early apoptosis rate of tumor cells was higher in the SFAD+5-FU and matrine+5-FU groups than the SFAD and matrine groups (*p* < 0.01) (Figures 6C–E,G).

### Effects of SFA/matrine+5-FU on serum cytokines related to cell proliferation and apoptosis

**FIGURE 7:**
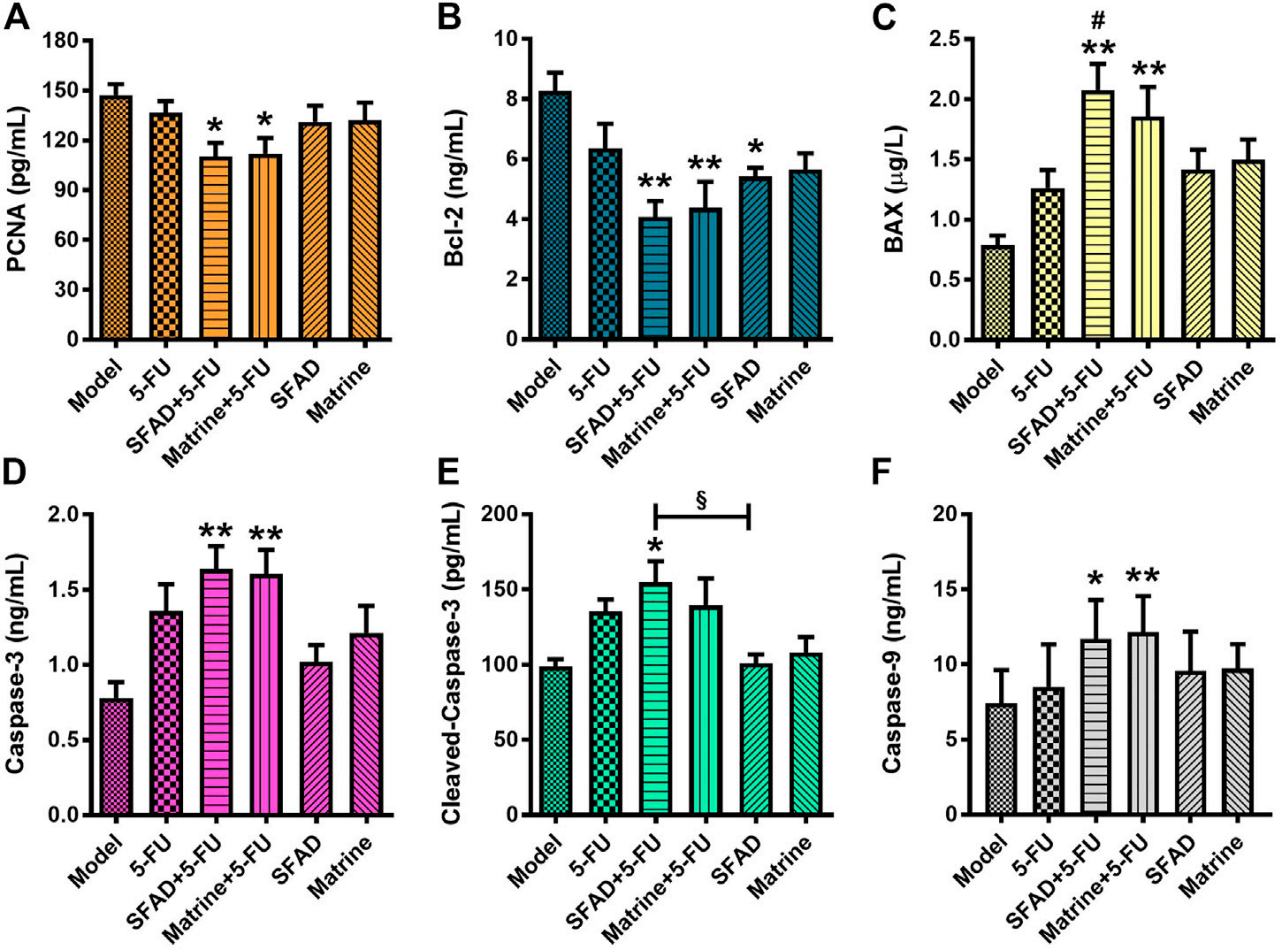
Effects of SFA/matrine+5-FU on serum proliferation/apoptosis-related factors in BGC-823-transplanted nude mice. **(A)** PCNA protein content in serum (pg/ml). **(B)** Bcl-2 protein content in serum (ng/ml). **(C)** BAX protein content in serum (μg/L). **(D)** Caspase-3 protein content in serum (ng/ml). **(E)** Cleaved Caspase-3 protein content in serum (pg/ml). **(F)** Caspase-9 protein content in serum (ng/ml). Data are presented as the mean ± SD of 7–8 nude mice. *p < 0.05, **p < 0.01 vs. the model group; ^#^ *p* < 0.05 vs. the 5-FU group; ^§^ *p* < 0.05 for the SFAD+5-FU vs. SFAD groups (one-way ANOVA).

The ELISA results showed that as compared with the normal saline group, both SFAD+5-FU and matrine+5-FU significantly decreased the serum levels of PCNA and Bcl-2, and increased the serum levels of Caspase-3, Caspase-9, and BAX (*p* < 0.05 and <0.01, respectively, Figures 7A–F). The single use of SFAD also reduced the serum content of Bcl-2 (*p* < 0.05), while SFAD+5-FU increased the serum content of cleaved Caspase-3 (*p* < 0.05, Figures 7B,E). Notably, SFAD+5-FU more effectively increased the serum content of BAX as compared with 5-FU alone and also increased the serum content of cleaved Caspase-3 more effectively than SFAD alone (*p* < 0.05, Figures 7C,E).

### Effects of SFA/matrine+5-FU on protein expression of MS4A10, MS4A8, MS4A7, PCNA, BAX, Bcl-2, Caspase-3, and cleaved Caspase-3 in tumor tissues

**FIGURE 8:**
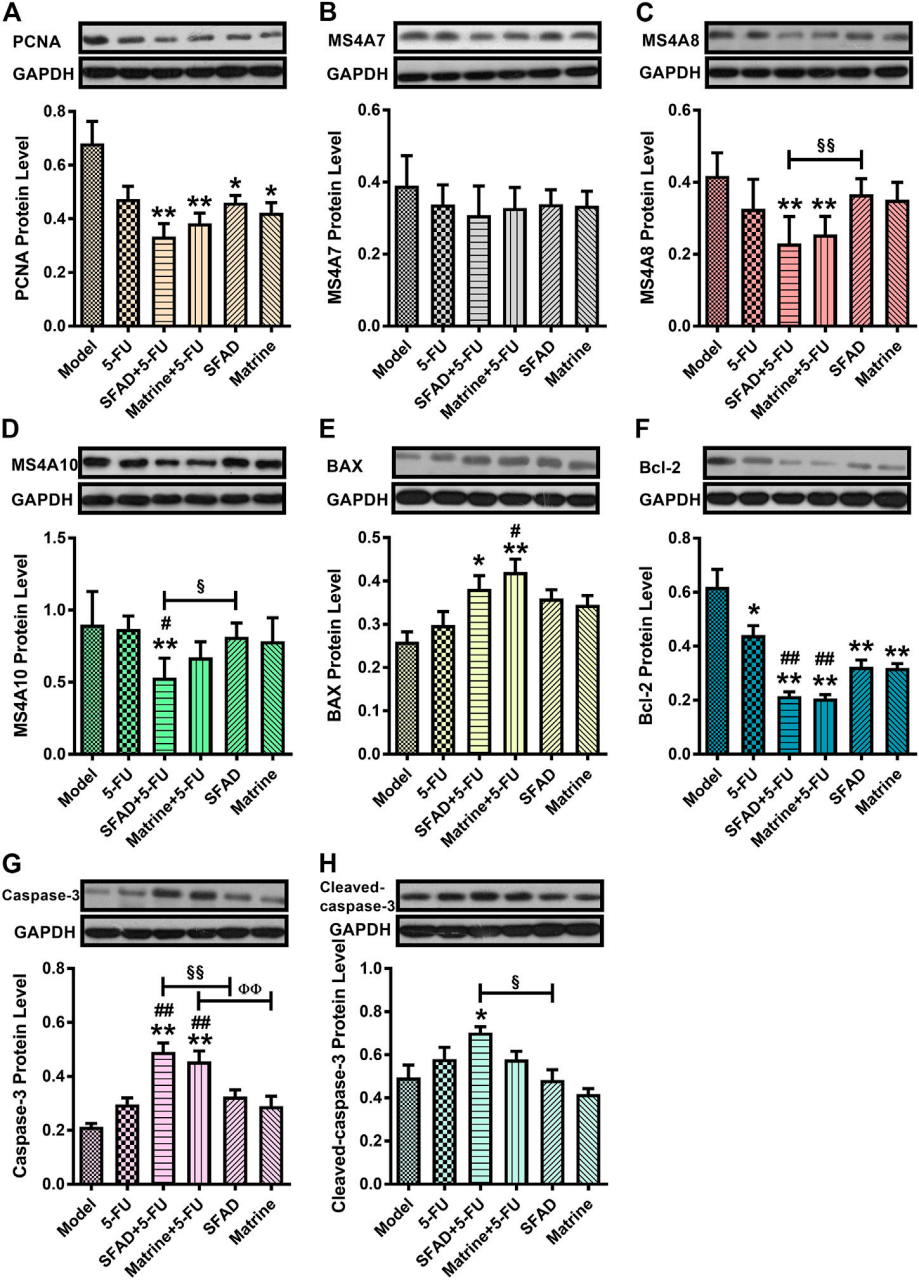
Effects of SFA/matrine+5-FU on serum protein expression of PCNA, MS4A7, MS4A8, MS4A10, Bax, Bcl-2, Caspase-3, and cleaved Caspase-8 in tumor tissues of BGC-823-transplanted nude mice. **(A)** Relative PCNA levels. **(B)** Relative MS4A7 levels. **(C)** Relative MS4A8 levels. **(D)** Relative MS4A10 levels. **(E)** Relative BAX levels. **(F)** Relative Bcl-2 levels. **(G)** Relative Caspase-3 levels. **(H)** Relative cleaved Caspase-3 levels. Data are presented as the mean ± SD of six nude mice per group. *p < 0.05, **p < 0.01 vs. the model group; ^#^ *p* < 0.05, ^##^ *p* < 0.01 vs. the 5-FU group; ^§^ *p* < 0.05, ^§§^ *p* < 0.01 for the SFAD+5-FU vs. SFAD groups; ^ΦΦ^ *p* < 0.01 for the matrine+5-FU vs. matrine groups (one-way ANOVA).

The results of western blot analysis showed that SFAD+5-FU treatment significantly reduced expression levels of the pro-proliferative proteins PCNA, MS4A8, and MS4A10, as well as the anti-apoptotic protein Bcl-2 in the tumor tissues of nude mice, and significantly increased expression levels of the pro-apoptotic proteins BAX, Caspase-3, and cleaved Caspase-3, as compared with the model group (*p* < 0.05 and <0.01, respectively, Figures 8A,C–H). Matrine+5-FU had almost the same curative effect as SFAD+5-FU. As compared with 5-FU alone, SFAD+5-FU significantly reduced expression of MS4A10, while SFAD+5-FU and matrine+5-FU decreased expression of Bcl-2 (*p* < 0.05 and <0.01, respectively, Figures 8D–G). Meanwhile, matrine+5-FU significantly increased expression of BAX and Caspase-3 (*p* < 0.05) and increased expression of Caspase-3 to a greater extent than matrine alone (*p* < 0.01). SFAD+5-FU reduced expression of MS4A8 and MS4A10 and increased expression of Caspase-3 and cleaved Caspase-3 to greater extents than SFAD alone (*p* < 0.05 and <0.01, respectively, Figures 8C,D,G,H). And compared with the model group, SFA/matrine+5-FU had a tendency to reduce the expression of MS4A7, but these differences were not significant (Figure 8B).

## Discussion

In China, most patients with GC are diagnosed with advanced disease or distant metastasis. For patients with advanced GC, systemic treatment (mainly chemotherapy) remains the first-line treatment strategy, as the development of chemotherapeutic agents for GC has entered a bottleneck stage, thus survival is relatively limited (Ding and Liang, 2020). As an important component traditional medicine, TCM has been widely used for treatment of GC for thousands of years. Syndrome differentiation and treatment based on the current overall state of patients can effectively improve symptoms, especially the side effects of radiotherapy and chemotherapy (Xie et al., 2019). Therefore, it is particularly important to actively explore TCM preparations to synergistically improve the effects of chemotherapeutic drugs.

In this study, in view of the fact that poorly differentiated human gastric carcinoma is more sensitive to anti-tumor drugs than well differentiated carcinoma (Greco et al., 1986; Maehara et al., 1987; Adachi et al., 2000), a subcutaneous transplanted tumor model of human GC BGC-823 cells (poorly differentiated) in nude mice was successfully established by the cell suspension planting method (Qiao et al., 2019) to study the effects of SFA or matrine alone and combined with low-dose 5-FU on the growth and apoptosis of transplanted GC cells. Different from previous studies in which high-dose 5-FU (150, 50, 30 mg/kg) was administered at one time or several times every few days as a control of chemotherapy (Aquino Esperanza et al., 2008; Park et al., 2019; Abulizi et al., 2021), this study adopted the chemotherapy mode of continuous administration of low-dose 5-FU (10 mg/kg). The results showed that SFA, matrine, and combinations with 5-FU inhibited the growth and proliferation of transplanted GC cells and promoted apoptosis, thereby confirming the therapeutic effects of TCM combined with 5-FU. In this experiment, 5-FU alone and combined with TCM had a better anti-tumor effect, but the obvious toxicity of chemotherapeutic drugs seriously affected the BW and growth of the mice. In contrast, SFA and matrine inhibited the growth of cancer cells and promoted a steady increase in BW of nude mice. These findings provide basic data for the role and mechanism of TCM combined with chemotherapy for the treatment of GC.

Numerous previous studies have found that adjuvant chemotherapy with TCM preparations can enhance cancer treatment and, thus, has become the focus of recent research (Liu et al., 2021; Lam et al., 2022). Paris saponin VII (PS VII) from *Trillium tschonoskii* maxim. Was reported to sensitize chemoresistant breast cancer MCF-7/ADR cells to the cytotoxic effects of adriamycin (Li et al., 2019). *Carthami Flos* (CF) and the dried flower of *Carthamus tinctorius* L. combined with other chemotherapeutic agents enhanced chemo-sensitivity by 2.8–4.0-fold and generally synergized the cytotoxic effect (Wu et al., 2013). Moreover, Tanshinone IIA combined with adriamycin synergistically inhibited the malignant biological behaviors of NSCLC A549 cells (Xie et al., 2016). The results of the present study showed that as compared with either TCM or chemotherapy alone, SFAD+5-FU more effectively promoted apoptosis and inhibited proliferation of GC cells, which confirmed the conclusions of previous studies that the effects of TCM were synergistic to those of chemotherapeutic agents.

Cell proliferation is the core event of tumor growth. Many important anti-tumor mechanisms inhibit cell proliferation *via* the cell cycle (Lemieszek et al., 2018; Gonsioroski et al., 2020). PCNA, a nuclear protein synthesized in G1 and S phases of the cell cycle, is used as a biomarker to monitor changes to cell growth (Mansilla et al., 2020). Regulation of PCNA expression is an important indicator of early changes to cell proliferation and provides a possible mechanism for SFA and chemotherapeutic drugs to inhibit cell proliferation. The results of this study confirmed that SFAD/matrine combined with 5-FU or SFA alone blocked the cell cycle of BGC-823 tumor cells in the G1/S phase, resulting in the failure of BGC-823 cells to divide and proliferate. MS4A is a newly described transmembrane gene family. As a cell surface signaling and intracellular adaptor protein, it also plays important roles in cell proliferation and cycle regulation (He et al., 2017). MS4A is abnormally expressed in GC, brain glioma, colon cancer, and other solid tumors and has been implicated in a variety of functions (Cruse et al., 2015; He et al., 2017; Sun et al., 2018; Zeng et al., 2022). MS4A2, MS4A6, MS4A7, MS4A8, MS4A14, and MS4A15 have been established as biomarkers of the progression and prognosis of GC (Sun et al., 2018). In addition, high expression of MS4A10 has been associated with the survival of GC patients (Yan et al., 2019). The results of the present study found that SFAD and matrine combined with 5-FU decreased the expression levels of PCNA, MS4A8, and MS4A10, indicating that the mechanism of SFAD+5-FU involved inhibition of the proliferative activity of PCNA and some MS4A proteins in GC cells. Moreover, SFAD+5-FU more effectively reduced MS4A8 expression than SFAD alone. However, at present, the mechanisms underlying the ability of MS4A family proteins to promote proliferation of tumor cells remain unclear, thus further studies are warranted.

The imbalance between the proliferation and apoptosis of cancer cells is closely related to the occurrence, metastasis, and prognosis of cancer (Krawczyk et al., 2014). A series of proto-oncogenes and tumor suppressor genes play regulatory roles in the process of apoptosis. Previous studies have shown that the tumor suppressor gene p53 plays important roles in cell growth, differentiation, apoptosis, aging, and other processes, including induction of cell cycle arrest, promotion of apoptosis, and DNA repair (Lieschke et al., 2019). Induction of apoptosis mainly activates key apoptotic factors through the death receptor-dependent, mitochondria-mediated apoptosis signaling pathway or by directly affecting the mitochondrial membrane through interactions with members of the Bcl-2 family of proteins (Hong and Mo, 2018). BAX has two functions: directly accelerating apoptosis and antagonizing the anti-apoptosis effect of Bcl-2 to a certain extent (Hassan et al., 2014). BAX generally exists in the cytoplasm as a monomer. When activated by apoptotic signals, BAX can change the permeability of the mitochondrial membrane, thereby promoting the release of cytochrome C and apoptosis-inducing factors, and activation of the caspase system, resulting in apoptosis (McKenna et al., 2021). Previous studies have shown that Caspase-9, as an initiator enzyme of apoptosis, activates the downstream effector enzyme Caspase-3 (Bratton and Salvesen, 2010; Molnár et al., 2021). Caspase-3 is one of the most important apoptosis executors in the caspase family and the main effector in the process of apoptosis. Activated Caspase-3 can induce chromosome division and is recognized as an indicator that apoptosis has entered an irreversible stage (Masood et al., 2020). In this study, expression levels of BAX, Caspase-3, Caspase-9, and cleaved Caspase-3 in the blood and tumor tissues of BGC-823-bearing nude mice were increased, while Bcl-2 expression was relatively low. SFAD+5-FU effectively reduced the protein content and expression of Caspase-3 and cleaved Caspase-3. These results suggest that the combination of SFAD or matrine with 5-FU induced activation of Caspase-3 protease to execute apoptosis of tumor cells *via* down-regulation of the apoptosis inhibitor Bcl-2 and up-regulation of BAX and Caspase-9.

The results of this study confirmed that SFA combined with 5-FU inhibited proliferation and promoted apoptosis of GC cells, thereby providing a theoretical and experimental basis for the clinical application of SFA and 5-FU for treatment of GC. However, there were some shortcomings in pharmacological elaboration. For example, the composition of SFA is complex, thus further research is needed to identify the specific metabolites underlying efficacy. Second, SFA combined with 5-FU has not been validated in other animal models. Third, other antitumor effects and mechanisms of SFA combined with 5-FU must be further explored. Fourth, the possibility of non-specific effects (artifacts) from single dose studies about SFA or matrine is also a major limitation.

## Conclusion

The combined administration of SFAD or matrine with 5-FU more effectively inhibited proliferation and induced apoptosis of GC cells than either drug alone, thereby confirming synergistic anti-GC effects. Although these effects need to be confirmed, they serve to underscore the potential value of SFA or matrine in combination with chemotherapy in the treatment of other malignant tumors in the future and encourage further research into this therapeutic modality.

## Data Availability

The original contributions presented in the study are included in the article/supplementary material, further inquiries can be directed to the corresponding authors.

## Abbreviations

BAX, Bcl-2 associated X protein; Bcl-2, B-cell leukemia/lymphoma 2; BW, body weight; ELISA, enzyme-linked immunosorbent assay; FBS, fetal bovine serum; 5-FU, 5-fluorouracil; GAPDH, glyceraldehyde-3-phosphate dehydrogenase; GC, gastric cancer; HE, hematoxylin and eosin; MS4A8, membrane-spanning 4-domains subfamily A member 8; MS4A7, membrane-spanning 4-domains subfamily A member 7; MS4A10, membrane-spanning 4-domains subfamily A member 10; PBS, phosphate-buffered saline; PCNA, proliferating cell nuclear antigen; SFA, *Sophora flavescens* Aiton; SFAD, *Sophora flavescens* Aiton decoction; TCM, traditional Chinese medicine; HPLC-MS/MS, high-performance liquid chromatography-tandem mass spectrometry.
